# Effects of Flutriafol Fungicide on the Lipid Accumulation in Human Liver Cells and Rat Liver

**DOI:** 10.3390/foods10061346

**Published:** 2021-06-10

**Authors:** Hyuk-Cheol Kwon, Hyejin Sohn, Do-Hyun Kim, Chang-Hee Jeong, Dong-Wook Kim, Sung-Gu Han

**Affiliations:** 1 Department of Food Science and Biotechnology of Animal Resources, Konkuk University, Seoul 05029, Korea; rnjs1024@konkuk.ac.kr (H.-C.K.); sonhjin123@konkuk.ac.kr (H.S.); secret311@konkuk.ac.kr (D.-H.K.); 2 Microbiology and Functionality Research Group, World Institute of Kimchi, Gwangju 61755, Korea; jeongch@wikim.re.kr; 3 Department of Poultry Science, Korea National College of Agriculture and Fisheries, Jeonju 54874, Korea; poultry98@korea.kr

**Keywords:** flutriafol, fungicide, pesticide residue, lipid accumulation, nonalcoholic fatty liver disease, oxidative stress, apoptosis

## Abstract

Flutriafol (FTF) is a triazole fungicide that can cause liver toxicity through the ingestion of its residues in food and water. However, little is known about the liver toxicity of FTF, particularly nonalcoholic fatty liver disease (NAFLD) in humans. Therefore, the purpose of this study was to investigate whether FTF induces NAFLD in human liver cells and animal liver. HepG2 cells and Sprague Dawley (SD) rats were treated with FTF at doses of 0–640 µM for 24 h and 0–150 mg/kg bw/day for 28 days, respectively. FTF (80, 160, and 320 µM) treatment to cells induced lipid accumulation. FTF (80 and 160 µM)-treated cells had higher levels of cytochrome P450 enzymes and reactive oxygen species and increased mitochondrial membrane potential loss than the control. FTF also increased the mRNA levels of antioxidant enzymes through oxidative stress and nuclear factor erythroid 2-related factor 2 pathways in HepG2 cells. However, a higher level of FTF (320 µM) induced apoptosis. The treatment of SD rats with FTF (2.5–150 mg/kg bw/day) induced fatty infiltration in the liver by impairing liver metabolism and inducing apoptosis. Therefore, our data suggest that human exposure to FTF residues may be a risk factor for liver diseases, such as NAFLD.

## 1. Introduction

Fungicides have been widely used in agriculture since the early 1900s to control fungal diseases and improve crop yield and quality [1,2]. The use of fungicides reduces global crop loss by approximately 20% [3]. Among the different types of fungicides, the azole group and triazoles have been commonly used since the 1970s to prevent or eradicate fungal infections in crops [1]. However, the overuse of fungicides causes the accumulation of fungicide residues in soil, groundwater, and crops [4]. Residual fungicides in crops are known to increase the risk of secondary poisoning in humans through ingested fungicide-contaminated foods [5].

Flutriafol (FTF), (RS)-2,4-difluoro-α-(1H-1,2,4-triazol-1-ylmethyl) benzyl alcohol, is a systemic triazole fungicide used to treat various grains and seeds [6]. FTF acts by interfering with the biosynthesis of ergosterol, which is essential for maintaining fungal cell membrane integrity by inhibiting cytochrome P450 51 (CYP51) [7,8]. However, according to the World Health Organization reports, short-term oral toxicity studies using mice, rats, and dogs have confirmed the hepatotoxicity of FTF, including liver weight gain, hepatocellular vacuolization, and lipid accumulation [9]. In addition, the European Food Safety Authority has reported that humans may be chronically exposed to residual FTF, which remains up to 0.5 mg/kg in crops and livestock [5]. Moreover, considering the acceptable daily intake of FTF (0.01 mg/kg bw/day), FTF has the potential to induce liver pathology in humans [5].

FTF absorbed through the gastrointestinal tract is transported to the liver through the hepatic portal vein. In the liver, the detoxification process occurs in three metabolic phases (phase I, II, and III) [10]. In particular, phase I metabolism is involved in oxidation, reduction, and hydrolysis, which promotes the excretion of xenobiotics by increasing their polarity [11,12]. It is known that CYP enzymes, such as CYP1A, 2B, 2C, and 3A, play a major role in phase I metabolism in the human liver. However, it has been revealed that these CYP enzymes can produce cellular reactive oxygen species (ROS), and the increased ROS levels can disrupt redox balance and induce oxidative stress [13].

Nonalcoholic fatty liver disease (NAFLD) is a chronic liver illness associated with metabolic syndrome, with a global prevalence rate of 25% [14,15]. Although the direct medical cost for NAFLD has been estimated to exceed 100 billion dollars in the United States alone due to devastating complications, there is no approved pharmacological treatment for NAFLD [16,17]. Insecticide exposure has been known to cause the development of NAFLD through the disruption of lipid metabolism [18]. Recent studies have shown that fungicides, such as cyproconazole, dazomet, fluazinam, hexaconazole, pyrasulfotole metabolite, and myclobutanil contributed to the pathogenesis of NAFLD [19,20]. Therefore, understanding the pathological mechanisms between fungicide and NAFLD is important for its prevention. A previous study reported that free thiol, a proxy of systemic oxidative stress through an imbalance of redox homeostasis, was associated with NAFLD progression and mortality [21]. Moreover, oxidative stress induced mitochondrial dysfunction and an unfolded protein response through endoplasmic reticulum stress [22,23]. Mitochondrial dysfunction and endoplasmic reticulum stress can lead to hepatocyte apoptosis through the intrinsic pathway in NAFLD patients. Thus, these previous data indicate that both oxidative stress and apoptosis play major roles in the progression of NAFLD.

Although there are risks of hepatotoxicity associated with human ingestion of residual FTF present in crops and water, the liver toxicity of FTF, such as NAFLD, and the underlying mechanisms have not been investigated. Therefore, the aim of this study was to investigate the risk of FTF on NAFLD development and progression, particularly lipid accumulation and the underlying cellular mechanisms using the human liver cell line HepG2 and a rat model.

## 2. Materials and Methods

### 2.1. Chemicals and Reagents

FTF, Pierce bicinchoninic acid protein assay kit, 4,6-diamidino-2-phenylindole dihydrochloride (DAPI), and 2, 2, 2-Tribromoethanol were obtained from Sigma-Aldrich (St. Louis, MO, USA). Dulbecco’s modified Eagle’s medium (DMEM), fetal bovine serum (FBS), penicillin/streptomycin, and 0.05% trypsin/0.53 mM ethylenediaminetetraacetic acid (EDTA) solution were provided by WELGENE Inc. (Gyeongsan, Daegu, Korea). Phosphate-buffered saline (PBS) was supplied by Lonza (Walkersville, MD, USA). T-25 flasks, 6-well plates, and 96-well plates were obtained from SPL Life Sciences (Pocheon, Gyeonggi, Korea). Dimethyl sulfoxide (DMSO) and N-acetylcysteine (NAC) were obtained from Amresco (Solon, OH, USA). The nitrocellulose membrane was supplied by GE Healthcare Bio-Sciences (Pittsburgh, PA, USA). Enhanced chemiluminescence was purchased from Thermo Fisher Scientific (Waltham, MA, USA) and Advansta (Menlo Park, CA, USA). Antibodies against nuclear factor erythroid 2-related factor 2 (Nrf2) were obtained from Abcam (Cambridge, MA, UK). Antibodies against Caspase-3 and B-cell lymphoma 2 (Bcl-2) were supplied by Cell Signaling Technology (Beverly, MA, USA). Antibodies against Bcl-2-associated X protein (Bax) and proliferating cell nuclear antigen (PCNA) were purchased from Santa Cruz Biotechnology Inc. (Santa Cruz, CA, USA). Antibodies against glyceraldehyde 3-phosphate dehydrogenase (GAPDH) were purchased from EMD Millipore (Burlington, MA, USA), and goat anti-rabbit IgG-HRP was obtained from Enzo Life Science (Farmingdale, NY, USA).

### 2.2. Cell Culture and Treatment

The human liver cell line HepG2 was obtained from the American Type Culture Collection. The cells were cultured in DMEM added with 10% FBS and 1% penicillin/streptomycin (*v*/*v*) in a humidified carbon dioxide (CO_2_) incubator supplied with 5% CO_2_ at 37 °C. The medium was replaced every 2 days and the cells were subcultured using 0.05% trypsin/0.53 mM EDTA solution. The cells were seeded in 6-well or 96-well plates at 4 days before FTF exposure (0–640 µM) or 0.004-0.016% DMSO (vehicle control) for 24 h.

### 2.3. Lactate Dehydrogenase (LDH) Activity Assay

Lactate dehydrogenase (LDH) release was evaluated using the Cytox 96^®^ Non-Radioactive Cytotoxicity Assay kit (Promega, Madison, WI, USA), according to the manufacturer’s protocol. HepG2 cells were seeded at a density of 1 × 10^4^ cells/well in 96-well plates and exposed to FTF (40, 80, 160, 320, and 640 µM) or DMSO (0.016%, vehicle control) for 24 h (*n* = 3 wells per group). Next, 10× lysis solution (10 µL per 100 µL) was added to the wells for determining maximum LDH release 45 min prior to the end of FTF treatment. Supernatants (50 µL) from the original 96-well plate were moved into a new 96-well plate, and a 50 µL CytoTox 96^®^ reagent was added to each well. After incubation for 30 min at room temperature in the dark, the stop solution (50 µL) was added to each well and the optical density (OD) was measured at 490 nm using an Epoch spectrophotometer (BioTek Instruments, Winooski, VT, USA). The released LDH percentage was calculated using the following formula:

LDH release (%) = (OD _Experimental_ LDH release)/(OD _Maximum_ LDH release) × 100 (1)

### 2.4. Cell Viability Assay

Cell viability was determined using 3-(4,5-dimethylthiazol-2yl)-2,5-diphenyl-2H-tetrazolium bromide (MTT; Amresco, Solon, OH, USA). HepG2 cells were seeded at a density of 1 × 10^4^ cells/well in 96-well plates and exposed to FTF (40, 80, 160, 320, and 640 µM) or DMSO (0.016%, vehicle control) for 24 h (*n* = 3 wells per group). The cells were replaced with fresh medium and then incubated with 10 µL MTT solution (5 mg/mL in PBS) at 37 °C for 3 h. After discarding 90 µL of the medium, 180 µL of acidic isopropanol was added to each well to solubilize the insoluble formazan crystals. Next, the 96-well plate was incubated at 37 °C for 1 h, and the OD was measured at 570 nm and 630 nm using an Epoch spectrophotometer. The OD at 630 nm was subtracted from the OD at 570 nm. The percentage of cell viability was calculated using the following formula:

Cell viability (%) = (OD _sample_/OD _control_) × 100 (2)

### 2.5. Oil Red O Staining

Intracellular neutral lipid such as triglycerides and cholesterol esters were evaluated using Oil red O dye (Sigma-Aldrich, St. Louis, MO, USA). HepG2 cells were seeded at a density of 2 × 10^5^ cells/well into 6-well plates and treated with 80, 160, and 320 µM FTF or 0.008% DMSO for 24 h (*n* = 3 wells per group). The cells were fixed with 10% formalin of 2.4 mL for 1 h at room temperature, and the fixed cells were rinsed with 60% isopropanol. Then, Oil red O staining solution of 1 mL was added to each well. After incubation for 10 min, the cells were washed with deionized distilled water thrice to remove the unbounded dye. Images were visualized and captured using a Nikon Eclipse Ti2-U and Nikon Elipse Ts2R camera (Nikon Co. Ltd., Tokyo, Japan). Then, the images were quantified using ImageJ software (National Institute of Health, Bethesda, MD, USA).

### 2.6. Measurement of Cellular Oxidative Stress

Intracellular ROS levels were estimated using 2,7-dichlorofluorescein diacetate (DCFDA; Sigma-Aldrich, St. Louis, MO, USA). HepG2 cells were grown in 6-well plates and exposed to 80 and 160 µM FTF or 0.004% DMSO (vehicle control) for 24 h with or without pretreatment with NAC (5 mM; in PBS) for 1 h (*n* = 3 wells per group). The cells were then treated with 10 µM DCFDA and incubated at 37 °C in a 5% CO_2_ incubator for 30 min. The cells were then washed thrice with PBS. The green fluorescence area stained with DCFDA was imaged using Nikon Eclipse Ti2-U and the fluorescence intensity was quantified using ImageJ software.

### 2.7. Measurement of Mitochondrial Membrane Potential

Mitochondrial membrane potential (MMP; ΔΨm) was evaluated using the JC-10 Mitochondrial Membrane Potential Assay Kit (Abcam, Cambridge, MA, USA), according to the manufacturer’s protocol. HepG2 cells were grown in Corning^®^ 96 Well Black Polystyrene Microplate (Sigma-Aldrich, St. Louis, MO, USA) and treated with 80 and 160 µM FTF or 0.004% DMSO (vehicle control) for 24 h (*n* = 3 wells per group). The cells were stained with 50 µL of JC-10 dye-loading solution. After incubation at room temperature for 45 min in the dark, 50 µL assay buffer B was added to each well. The integrity of MMP was determined as the ratio of the fluorescence of JC-10 aggregates (Ex/Em = 490/525 nm) to monomeric JC-10 (Ex/Em = 540/590 nm). The fluorescence intensity was determined using a SpectraMax Gemini EM Microplate Reader (Molecular Devices, CA, USA). The fluorescence ratio was calculated using the following formula:

Fluorescence ratio (%) = (Fluorescence _525 nm_/Fluorescence _590 nm_) × 100 (3)

### 2.8. Determination of the Gene Expression Levels of Cytochrome P450 and Antioxidant Enzymes

The gene expression levels of CYP enzymes and antioxidant enzymes were determined using real-time polymerase chain reaction (RT-PCR). HepG2 cells were grown in 6-well plates and then treated with 80 and 160 µM FTF or 0.004% DMSO (vehicle control) for 24 h with or without pretreatment with NAC (5 mM; in PBS) for 1 h (*n* = 3 wells per group). RNA was obtained from the cells using TRIzol reagent (Ambion, Austin, TX, USA). Reverse transcription was conducted using the TOPscript RT DryMIX kit (Enzynomics, Daejeon, Korea) to synthesize cDNA. The mRNA expression level was estimated by using an RT-PCR system (Roche LightCycler^®^ 96 System, Basel, Switzerland) with a total volume of 20 µL of PCR reaction mixture containing 10 µL of 2× Real-Time PCR Smart mix (Solgent, Daejeon, Korea), 8 µL of RNase-free water, 1 µL of cDNA sample, and 0.5 µL each of 10 µM forward and reverse primers (Bionics, Seoul, Korea). The thermal condition of PCR reaction consisted of 60 cycles of 95 °C for 15 min, followed by 60 cycles of denaturation at 95 °C for 10 s, annealing at 60 °C for 10 s, and extension at 72 °C for 10 s. The mRNA expression level was quantified by the 2^−ΔΔCt^ method, and GAPDH was used as an internal control. Primer sequences were designed using the AmplifX software (Table 1).

### 2.9. Determination of Nuclear Translocation of Nrf2 Using Nuclear Fractionation and Immunofluorescence Microscopy

Nuclear translocation of Nrf2 was determined by nuclear fractionation and immunofluorescence staining. HepG2 cells were grown in 6-well plates and exposed to 160 µM FTF or 0.004% DMSO (vehicle control) for 24 h with or without pretreatment with NAC (5 mM; in PBS) for 1 h (*n* = 3 wells per group). Cells were lysed in hypotonic buffer containing 20 mM Tris (pH 7.4), 10 mM sodium chloride (NaCl), 3 mM magnesium chloride (MgCl_2_), and protease inhibitor mixture (Abbkine, Wuhan, China). Next, 10% Triton X-100 was added to extract the cytosolic proteins, and the supernatants were eliminated through centrifugation at 3000× *g* for 10 min at 4 °C. The cell pellets were resuspended in cell extraction buffer containing 100 mM Tris (pH 7.4), 2 mM sodium orthovanadate (Na_3_VO_4_), 100 mM NaCl, 1% Triton X-100, 1 mM EDTA, 10% glycerol, 0.1% SDS, 20 mM tetrasodium pyrophosphate (Na_4_P_2_O_7_), and a protease inhibitor mixture. The homogenates were centrifuged at 14,000× *g* for 20 min at 4 °C, and the supernatants were used for Western blotting. For immunofluorescence staining, HepG2 cells were grown in 6-well plates and exposed to 160 µM FTF or 0.004% DMSO (vehicle control) for 24 h with or without pretreatment with NAC (5 mM; in PBS) for 1 h (*n* = 3 wells per group). The cells were fixed with 4% paraformaldehyde for 15 min and then permeabilized using 0.1% Triton X-100 in PBS for 10 min. Next, a blocking buffer consisting of 3% bovine serum albumin and 2% normal donkey serum was used to block the cell monolayers, and the cells were incubated with anti-Nrf2 diluted in blocking buffer at 4 °C for 24 h. After washing with PBS, the cells were incubated with DyLight^TM^ -488-conjugated anti-IgG at room temperature for 1 h and washed thrice with PBS, followed by fixation with 4% paraformaldehyde. Subsequently, the cell nuclei were stained with DAPI (1 µg/mL) for 30 min and washed thrice with PBS. The fluorescence areas were photographed using a Nikon Eclipse Ti2-U microscope.

### 2.10. Determination of Apoptosis Using Annexin V/Propidium Iodide Assay

Cell death was determined using the EzWay Annexin V-FITC Apoptosis Detection Kit (Sigma-Aldrich, St. Louis, MO, USA) according to the manufacturer’s instructions. HepG2 cells were grown in 6-well plates and exposed to 320 µM FTF or 0.008% DMSO (vehicle control) for 24 h with or without pretreatment with NAC (5 mM; in PBS) for 1 h (*n* = 3 wells per group). The medium was collected in a 15 mL tube after the end of the FTF treatment, and the cells were washed twice with PBS. Next, the cells were harvested and pelleted by centrifugation at 3000× *g* for 5 min at 4 °C. Subsequently, the cells were washed with 4 °CPBS and resuspended in 1× binding buffer. Next, the cells were stained with Annexin V-fluorescein isothiocyanate (1.25 µL) and propidium iodide (10 µL) and incubated in the dark for 10 min at room temperature. The stained samples were analyzed using a CytoFlex Flow Cytometry Analyzer (Beckman Coulter, Brea, CA, USA).

### 2.11. Animal Experiments

The animal experiments were carried out in accordance with the Institutional Animal Care and Use Committee (IACUC) of the Ethics Committee of Konkuk University (Seoul, Korea; IACUC No. KU20176), and the animals were treated humanely to alleviate distress and discomfort. Eight-week-old female Sprague Dawley (SD) rats (220–250 g) were purchased from Orient Bio Inc. (Seongnam, Gyeonggi, Korea) and acclimatized for 1 week. The rats were nurtured under controlled constant temperature (22–25 °C) and humidity (40–70%) with 12 h light–dark cycles. All the animals were provided free access to feed and water ad libitum. Six groups of 30 animals each were used in this study: (1) FTF 0 mg/kg bw/day (*n* = 5); (2) FTF 0.25 mg/kg bw/day (*n* = 5); (3) FTF 2.5 mg/kg bw/day (*n* = 5); (4) FTF 25 mg/kg bw/day (*n* = 5); (5) FTF 75 mg/kg bw/day (*n* = 5); (6) FTF 150 mg/kg bw/day (*n* = 5). The dose of FTF (2.5 mg/kg bw/day) for rats was determined with the consumer chronic risk value in agriculture commodities (0.5 mg/kg) using body surface area (BSA) [21]. Body surface area was calculated using the following formula: Rat FTF dose (mg/kg) = Human FTF input value (mg/kg) multiplied by [Human Km (37)/Rat Km (6)]. FTF was diluted in corn oil and administered to rats for 28 days through oral gavage. The rats were anesthetized by an i.p. injection of 300 mg/kg avertin. Subsequently, liver and kidney samples from the rats were washed with saline, and the weights of the organs were measured. Liver tissues were divided into two parts: the left and right lobes. The left lateral lobes were preserved at −80 °C for Western blotting and RT-PCR analysis. The right lateral lobes were fixed in 10% formalin for histopathological examination.

### 2.12. Biochemical Analysis of Blood Plasma

The biochemical profiles of the plasma samples were analyzed using an automated dry chemistry analyzer (FUFI DRI-CHEM 7000i; Fujifilm Corp, Tokyo, Japan). Blood was collected into BD Vacutainer^®^ K2EDTA tubes (Becton Dickinson and Company, Sparks, USA) through cardiac puncture. Blood samples were centrifuged at 3000× *g* for 10 min at 4 °C, and the supernatants were stored at –80 °C for further experiments. Aspartate aminotransferase (AST), alkaline phosphatase (ALP), alanine aminotransferase (ALT), albumin (ALB), urea nitrogen (BUN), and total protein (TP) were evaluated as biochemical parameters.

### 2.13. Histological Analysis

Liver samples were collected and fixed in 10% formalin for histopathological analysis. Thereafter, the tissue samples were dehydrated in ascending grades of ethanol solution and de-waxed with xylene. The tissue pieces were cut into 3.5 µm sections using a microtome. Next, the sections were stained with hematoxylin and eosin, and histological alterations were mainly examined for fatty infiltration. The slides were photographed using a Nikon Eclipse Ti2-U and Nikon Eclipse Ts2R camera.

### 2.14. Determination of Protein Expression Level of Nrf2 and Apoptosis-Related Markers Using Western Blot Analysis

Protein expressions of Nrf2 and apoptosis-related markers were determined by Western blotting. Rat liver samples stored at −80 °C were immersed in liquid nitrogen and crushed for further experiments. Next, the liver tissue protein (0.01 g) was lysed using a 1 mL radioimmunoprecipitation assay buffer (Elpis Biotech, Daejeon, Korea) containing a protease inhibitor cocktail. The tissue lysates were sonicated at 30 kHz using a probe sonicator (Qsonica Q55 Sonicator, Newtown, NW, USA) and centrifuged at 18,000× *g* for 20 min at 4 °C to eliminate tissue debris. The supernatants were analyzed using a bicinchoninic acid protein assay kit. Western blot protein samples (20 µg) were separated by sodium dodecyl sulfate–polyacrylamide gel electrophoresis and transferred to a nitrocellulose membrane. The membrane was blocked with 3% nonfat milk buffer for 1.5 h at room temperature and incubated with primary antibodies overnight at 4 °C. The membrane was washed thrice with Tris-buffered saline with Tween 20 and then incubated with horseradish peroxidase-conjugated secondary antibody for 1.5 h at room temperature. The protein bands were visualized by enhanced chemiluminescence Western blot detection reagents and quantified using ImageJ software. In addition, the nitrocellulose membrane was used again after the stripping process as follows: the nitrocellulose membrane was incubated in stripping buffer for 30 min at 57 °C. The membrane was washed thrice with distilled water for 5 min, followed by three washes with Tris-buffered saline with Tween 20 for 10 min. Subsequently, the membrane was blocked again for Western blot analysis.

### 2.15. Statistical Analysis

The experimental data are presented as mean ± standard error of the mean (SEM). Statistical significance was analyzed using one-way ANOVA with post hoc Dunnett’s test and Student’s t-test. The tests were performed using SPSS-PASW statistics software version 18.0 for Windows (SPSS, Chicago, IL, USA). Statistical difference was determined at *P* < 0.05.

## 3. Results

### 3.1. Flutriafol-Induced Cell Damage, Reduced Cell Viability, and Lipid Accumulation in Human Liver Cells

**Figure 1:**
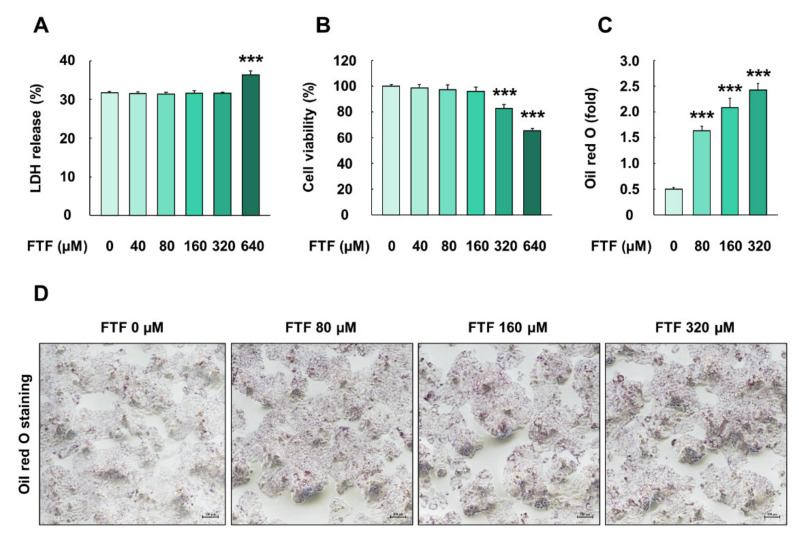
Effects of FTF on LDH release, cell viability, and lipid accumulation in HepG2 cells: (**A**) LDH release and (**B**) cell viability in HepG2 cells. The cells were treated with FTF (0, 40, 80, 160, 320, and 320 µM for 24 h (*n* = 3 wells per group); (**C**) quantification of red area and (**D**) Image in HepG2 cells stained with Oil red O The magnification of the image was 100×. The cells were treated with FTF at the concentration of 80, 160, and 320 µM for 24 h (*n* = 3 wells per group). Images are representative of three independent experiments. The results are expressed as mean ± SEM and statistically significant difference, as compared to the control (DMSO), is indicated as *** *P* < 0.001. Abbreviations: FTF, flutriafol; LDH, lactate dehydrogenase; MTT, 3-(4,5-dimethylthiazole-2yl)-2,5-diphenyltetrazolium bromide; SEM, standard error of mean; DMSO, dimethyl sulfoxide.

The cells treated with FTF at a concentration of 640 µM for 24 h had significantly higher LDH release levels than the control cells (*P* < 0.001; Figure 1A). In addition, the exposure of cells to 320 and 640 µM FTF for 24 h caused a significant decrease in cell viability, up to 83% and 65%, compared with the control cells, respectively (*P* < 0.001; Figure 1B). Thus, FTF at concentrations of 80, 160, and 320 µM was used for further experiments. FTF at a concentration of 640 µM was not used in further experiments because cell membrane damage had occurred. Moreover, FTF treatment to cells induced prominent lipid accumulation in the cytoplasm, compared with the control cells (*P* < 0.001; Figure 1C,D).

### 3.2. Flutriafol-Induced Cytochrome P450 Activation in Human Liver Cells

**Figure 2:**
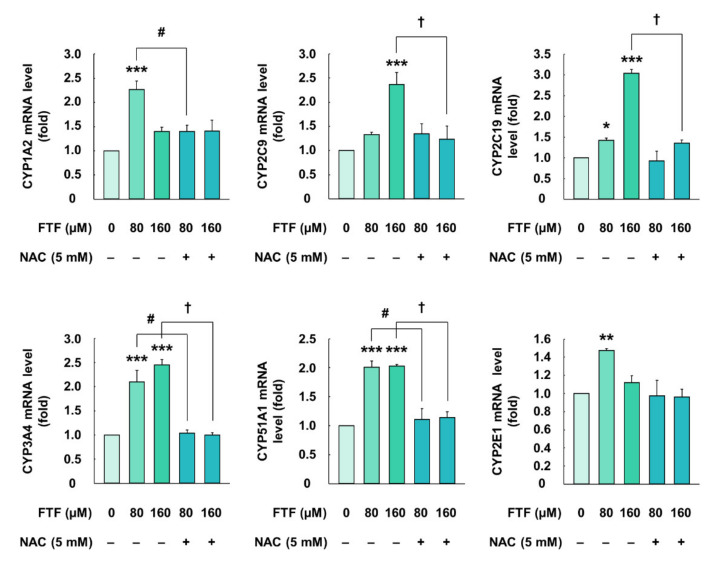
Effects of FTF on gene expression of CYP1A2, 2C9, 2C19, 3A4, 51A1, and 2E1 in HepG2 cells. The cells were treated with 80 and 160 µM FTF for 24 h with 5 mM NAC pretreatment for 1 h (*n* = 3 wells per group). GAPDH was used as the housekeeping gene. The results are expressed as mean ± SEM and statistically significant differences, compared to the control (DMSO), are indicated as * *P* < 0.05, ** *P* < 0.01, and *** *P* < 0.001. # *P* < 0.05 and † *P* < 0.05 indicate a significant difference, compared to the 80 and 160 µM FTF treatment groups, respectively. Abbreviations: FTF, flutriafol; CYP, cytochrome P450; NAC, N-acetylcysteine; GAPDH, glyceraldehyde 3-phosphate dehydrogenase; SEM, standard error of mean; DMSO, dimethyl sulfoxide.

The cells treated with FTF (80 and 160 µM, for 24 h) had significantly higher mRNA expression of CYP1A2, CYP2C9, CYP2C19, CYP3A4, CYP51A1, and CYP2E1 than control cells (*P* < 0.05; Figure 2). However, the cells treated with the antioxidant NAC (5 mM, for 1 h) had significantly lower mRNA expression of CYPs than those treated with FTF alone (*P* < 0.05). These results showed a correlation between the activation of CYP and the generation of cellular oxidative stress in human liver cells.

### 3.3. Flutriafol-Induced Oxidative Stress and Mitochondria Membrane Potential in Human Liver Cells

**Figure 3:**
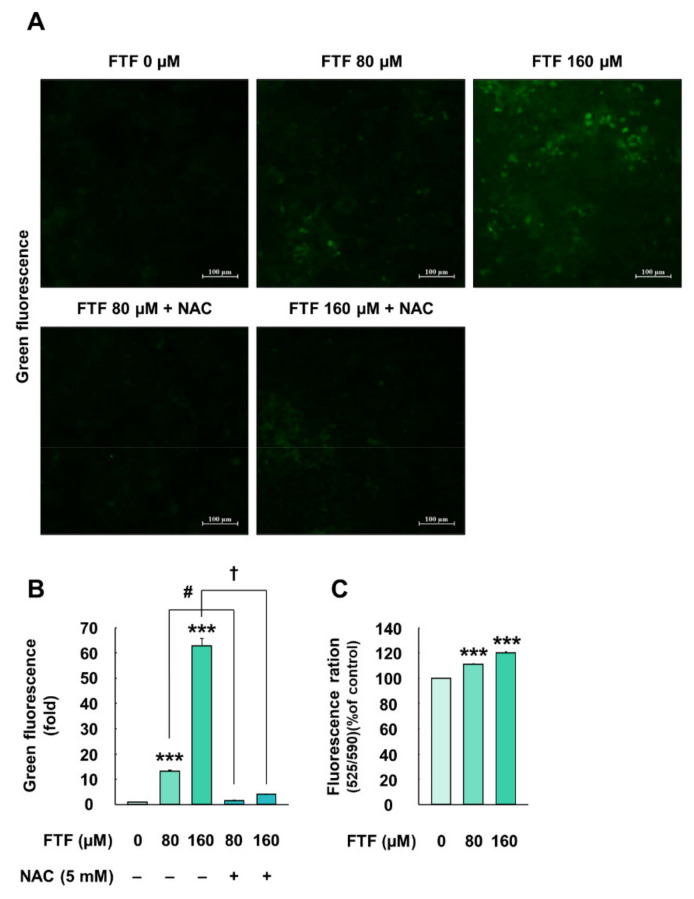
Effects of FTF on cellular ROS production and mitochondria membrane potential in HepG2 cells: (**A**) fluorescence image of ROS generation and (**B**) quantification of the green fluorescence area in HepG2 cells. The magnification of the image was 200×. The cells were treated with 80 and 160 µM FTF for 24 h with 5 mM NAC pretreatment for 1 h (*n* = 3 wells per group); (**C**) fluorescence ratio of JC-10 monomeric form (525 nm) to JC-10 aggregate form (590 nm) in HepG2 cells. The magnification of the image was 200×. The cells were treated with 80 and 160 µM FTF for 24 h (*n* = 3 wells per group). Images are representative of three independent experiments. The results are expressed as the mean ± SEM and statistically significant difference, as compared to the control (DMSO), is indicated as *** *P* < 0.001. # *P* < 0.05 and † *P* < 0.05 indicate a significant difference, compared to the 80 and 160 µM FTF treatment group, respectively. Abbreviations: ROS, reactive oxygen species; FTF, flutriafol; NAC, N-acetylcysteine; SEM, standard error of mean; DMSO, dimethyl sulfoxide.

HepG2 cells exposed to 80 and 160 µM FTF for 24 h showed 13.13- and 62.83-folds higher levels of intracellular ROS, respectively, compared with the control cells (*P* < 0.001; Figure 3A,B). However, pretreatment with NAC (5 mM, for 1 h) improved the intracellular ROS levels (1.49-and and 4.01-fold, respectively, compared with the control; Figure 3A,B). In addition, HepG2 cells treated with FTF had a significantly higher fluorescence ratio of mitochondrial membrane potential, compared with the control cells (*P* < 0.001; Figure 3C).

### 3.4. Flutriafol-Induced Intracellular Signaling Pathway and Antioxidant Enzymes in Human Liver Cells

**Figure 4:**
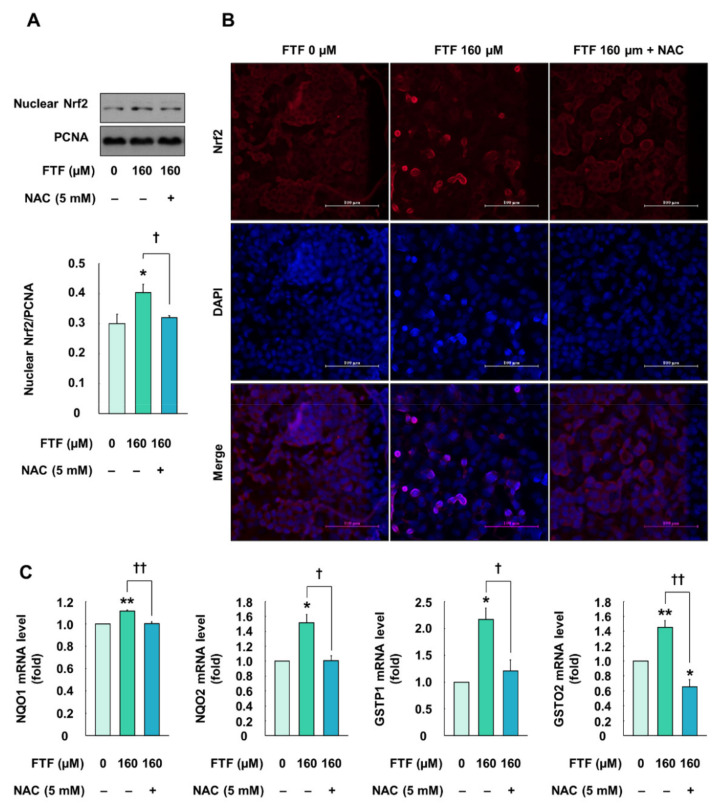
Effects of FTF on Nrf2 translocation and gene expression of antioxidant enzymes in HepG2 cells: (**A**) nuclear translocation and (**B**) fluorescence image of Nrf2 in HepG2 cells. The magnification of the image was 400×; (**C**) gene expression of antioxidant enzymes, including NQO1, NQO2, GSTP1, and GSTO2 in HepG2 cells. The cells were treated with 160 µM FTF for 24 h with 5 mM NAC pretreatment for 1 h (*n* = 3 wells per group). PCNA and GPADH were used as housekeeping protein and gene. Images are representative of three independent experiments. The results are expressed as mean ± SEM and statistically significant differences, as compared with the control (DMSO), are indicated as * *P* < 0.05 and ** *P* < 0.01. † *P* < 0.05 and †† *P* < 0.01 indicate a significant difference, compared with the 160 µM FTF treatment group. Abbreviations: FTF, flutriafol; Nrf2, nuclear factor erythroid 2-related factor 2; NAC, N-acetylcysteine; NQO, NAD(P)H quinone oxidoreductase; GSTP, glutathione S-transferase P; GSTO, glutathione S-transferase omega; PCNA, proliferating cell nuclear antigen; GAPDH, glyceraldehyde 3-phosphate dehydrogenase; SEM, standard error of mean; DMSO, dimethyl sulfoxide.

Cells treated with FTF (160 µM, for 24 h) showed significantly increased transfer of Nrf2 from the cytosol to the nucleus, compared with the control cells (*P* < 0.05; Figure 4A). However, cells pretreated with NAC (5 mM, for 1 h) showed a decreased nuclear translocation, compared with FTF alone-treated cells (*P* < 0.05). Furthermore, the fluorescence microscopy images were confirmed that Nrf2 proteins were translocated from the cytosol to the nucleus in FTF-treated cells, which was reversed by NAC treatment (Figure 4B). Therefore, the expression levels of antioxidant enzyme genes associated with Nrf2 were determined in HepG2 cells. Treatment of cells with FTF (160 µM, for 24 h) significantly increased mRNA expression of NAD(P)H quinone oxidoreductase (NQO) 1, NQO2, glutathione S-transferase (GSTP) 1, and glutathione S-transferase omega (GSTO) 2 (*P* < 0.05; Figure 4C). However, the cells pretreated with NAC (5 mM, for 1 h) had significantly lower mRNA expression levels of these antioxidant enzymes than the cells exposed to FTF alone (*P* < 0.05).

### 3.5. Flutriafol-Induced Apoptosis in Human Liver Cells

**Figure 5:**
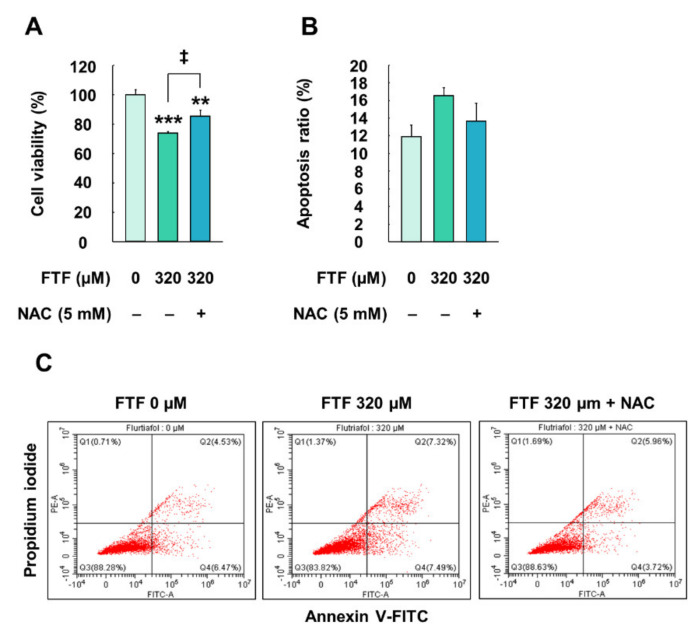
Effects of FTF on cell viability and apoptosis in HepG2 cells: (**A**) cell viability in HepG2 cells; (**B**) quantification of the apoptotic cell ratio (early and late apoptosis) and (**C**) the percentages of necrotic (Q1), late apoptotic (Q2), viable (Q3), and early apoptotic (Q4) in HepG2 cells. The cells were treated with 320 µM FTF for 24 h with 5 mM NAC pretreatment for 1 h (*n* = 3 wells per group). Images are representative of three independent experiments. The results are expressed as mean ± SEM, and statistically significant differences, compared with the control (DMSO), are indicated as ** *P* < 0.05 and *** *P* < 0.001. ‡ *P* < 0.05 indicates a significant difference, compared to the 320 µM FTF treatment group. Abbreviations: FTF, flutriafol; NAC, N-acetylcysteine; SEM, standard error of mean; DMSO, dimethyl sulfoxide.

HepG2 cells exposed to FTF (320 µM, for 24 h) showed significantly lower cell viability than the control cells (*P* < 0.05; Figure 5A). However, NAC (5 mM, for 1 h)-pretreated cells had significantly higher cell viability than the cells treated with FTF (*P* < 0.05). Furthermore, the flow cytometry data displayed that the apoptotic ratio (early apoptotic and late apoptotic cells) increased after treatment with 320 µM FTF for 24 h (Figure 5B,C). This apoptotic ratio decreased to the control level in the cells pretreated with NAC, compared with cells treated only with FTF.

### 3.6. Flutriafol-Induced Changes in Body Weight, Feed Intake, Organ Weight/Body Weight Ratio, and Biochemical Profiles in Rats

**Figure 6:**
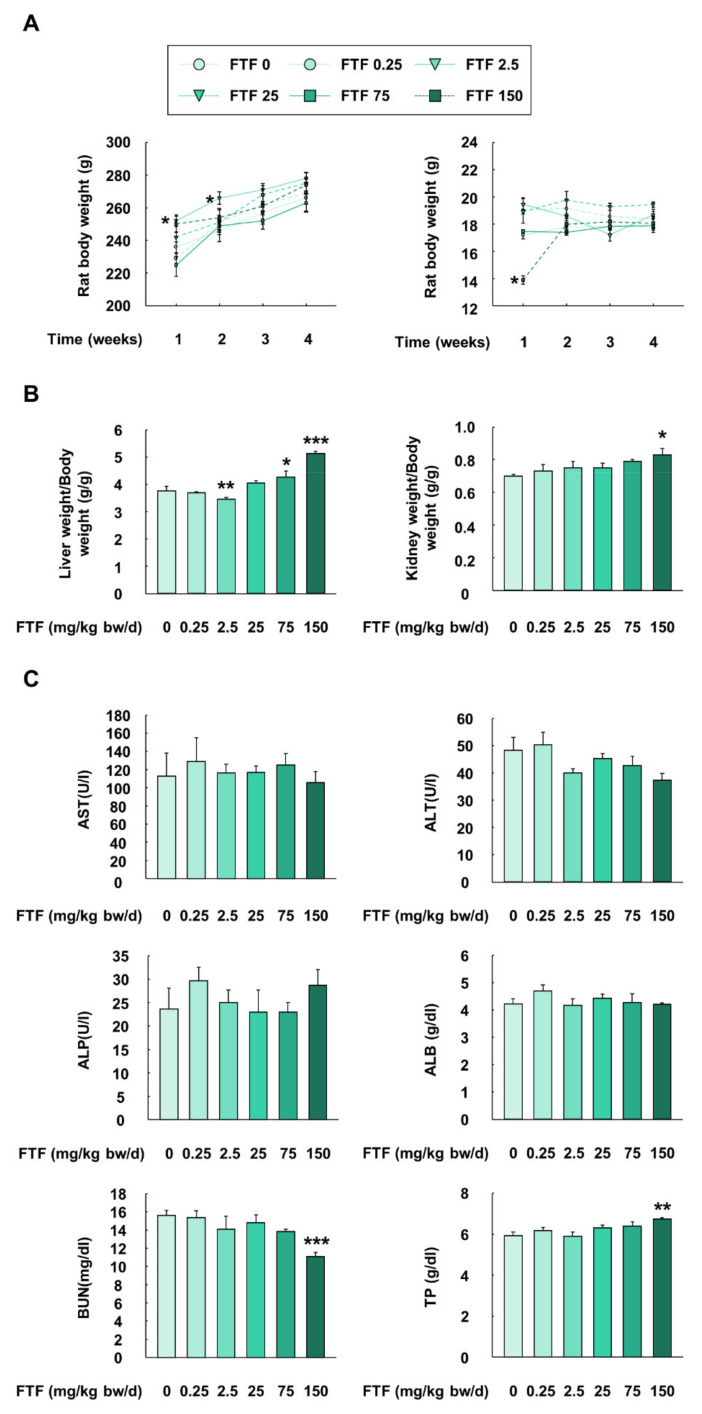
Effects of FTF on body weight, feed intake, organ weight, and biochemical profiles in female SD rats: (**A**) the change of body weight and feed intake in female SD rat; (**B**) liver weight/body weight and kidney weight/body weight in female SD rats; (**C**) plasma biochemical profiles including AST, ALT, ALP, ALB, BUN, and TP in female SD rats. Rats were administered FTF at 0, 0.25, 2.5, 25, 75, and 150 mg/kg bw/day for 28 days (*n* = 3 animals per group). The results are expressed as mean ± SEM and statistically significant differences, as compared to the control (corn oil), are indicated as * *P* < 0.05, ** *P* < 0.01, and *** *P* < 0.001. Abbreviations: AST, aspartate aminotransferase; ALT, alanine aminotransferase; ALP, alkaline phosphatase; ALB, albumin; BUN, urea nitrogen; TP, total protein; SD, Sprague Dawley; FTF, flutriafol; SEM, standard error of mean.

An animal study was performed with rats to provide further evidence for FTF-induced liver metabolism and apoptosis. Although the rats treated with FTF (2.5 mg/kg bw/day) had significantly higher body weight than control rats at 1 and 2 weeks (*P* < 0.05), there was no statistically significant difference in body weight among the treatment groups from 3 weeks (Figure 6A). In addition, although the rats treated with 150 mg/kg bw/day FTF had significantly lower levels of feed intake than the control rats (*P* < 0.05), the feed intake levels recovered after 1 week (Figure 6A). The rats treated with 75 and 150 mg/kg bw/day FTF had a significantly increased liver weight/body weight ratio than the control rats (*P* < 0.05; Figure 6B). The rats treated with 75 and 150 mg/kg bw/day FTF had significantly increased kidney weight/body weight ratio than the control rats (*P* < 0.05). Regarding biochemical parameters, there were no significant differences in the rats treated with FTF (0–150 mg/kg bw/day) and the control rats, with regard to the plasma levels of AST, ALT, ALP, and ALB (Figure 6C). However, the rats treated with the highest dose of FTF (150 mg/kg bw/day) had a significantly lower level of BUN and a higher level of TP than the control rats (*P* < 0.01).

### 3.7. Flutriafol-Induced Activation of Cytochrome P450 and Nrf2 Signaling in Rat Liver

**Figure 7:**
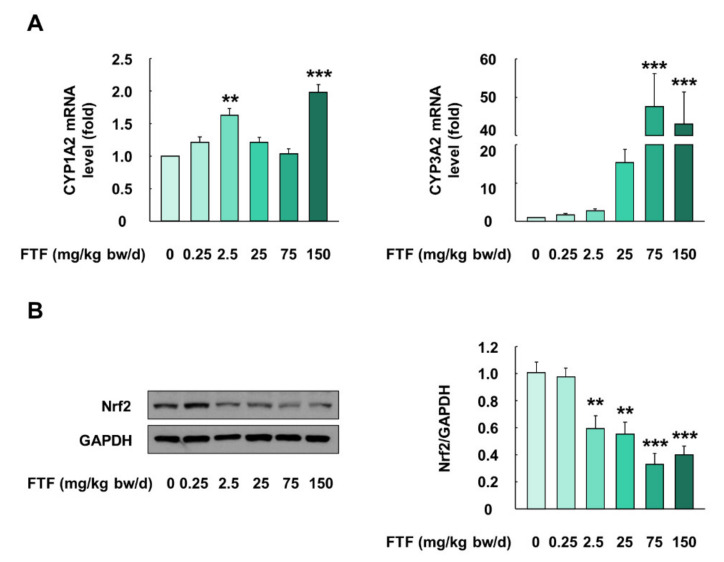
Effects of FTF on gene expression of CYP enzymes and Nrf2 protein expression in female SD rat liver: (**A**) gene expression of CYP1A2 and 3A2 in female SD rat liver; (**B**) protein expression of Nrf2 in female SD rat livers. Rats were administered FTF at 0, 0.25, 2.5, 25, 75, and 150 mg/kg bw/day for 28 days (*n* = 3 animals per group). GAPDH was used as the housekeeping protein and gene. Images are representative of three independent experiments. The results are expressed as mean ± SEM and statistically significant differences, as compared to the control (corn oil), are indicated as ** *P* < 0.01 and *** *P* < 0.001. Abbreviations: CYP, cytochrome P450; Nrf2, nuclear factor erythroid 2-related factor 2; SD, Sprague Dawley; FTF, flutriafol; GAPDH, glyceraldehyde 3-phosphate dehydrogenase; SEM, standard error of mean.

FTF-treated rats showed changes in organ weight/body weight ratio and biochemical profile, and hence, metabolism studies related to FTF were conducted using rat liver (Figure 7A,B). Liver samples collected from FTF-treated rats (0–150 mg/kg bw/day) showed higher mRNA expression levels of CYP1A2 and CYP3A2 than those of control rats (*P* < 0.01; Figure 7A). In particular, the expression level of CYP3A2 was markedly higher than that of CYP1A2 (Figure 7A). Furthermore, the liver samples collected from FTF-treated rats (2.5–150 mg/kg bw/day) had a significantly lower protein level of Nrf2 than those from the control rats (*P* < 0.01; Figure 7B).

### 3.8. Flutriafol-Induced Apoptosis in Rat Liver

**Figure 8:**
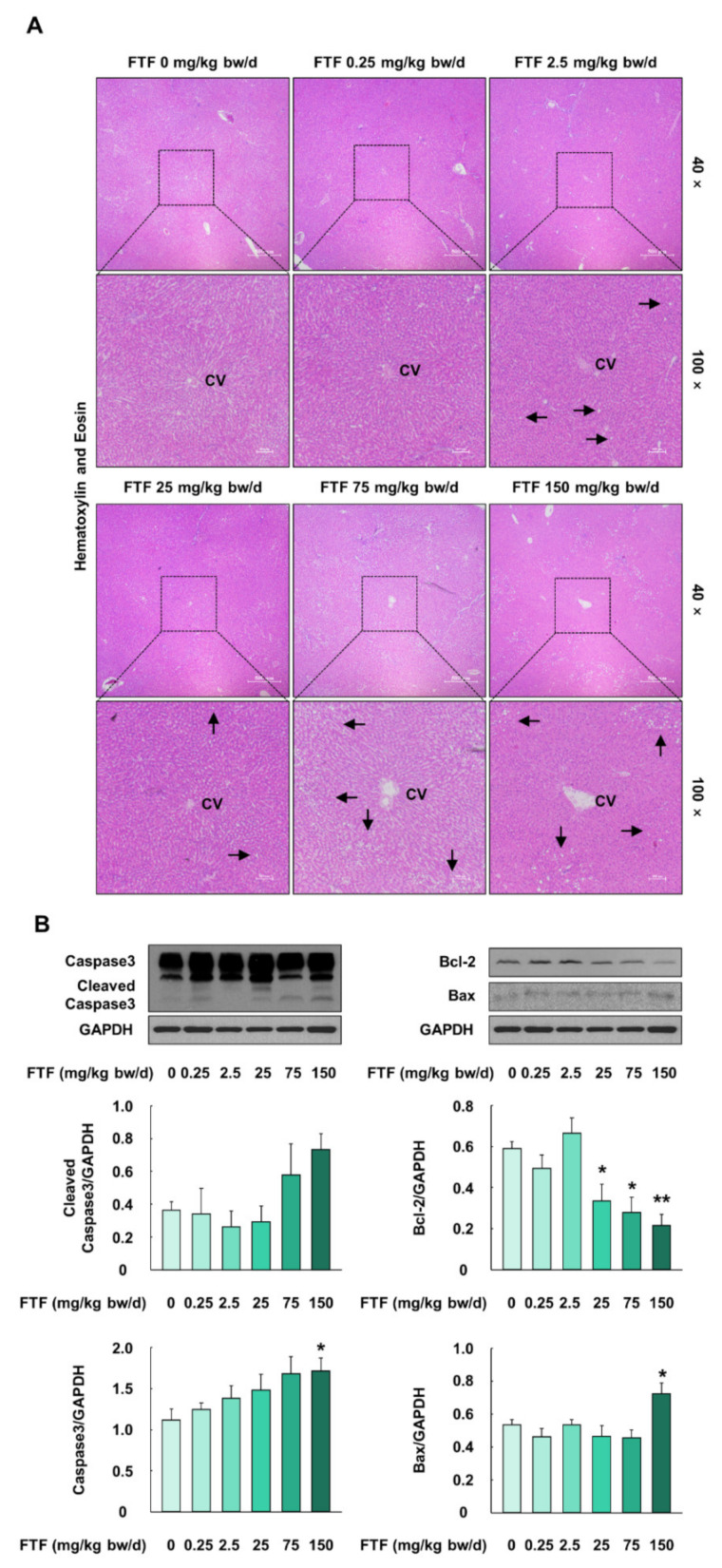
Effects of FTF on histological alterations and apoptosis markers in rat liver: (**A**) hematoxylin and eosin staining of rat liver sections. The magnification of the image was 40× and 100×; (**B**) protein expression of Caspase3, cleaved-Caspase3, Bcl-2, and Bax in female SD rat livers. Black arrows indicate fatty infiltration in rat liver. Rats were administered FTF at 0, 0.25, 2.5, 25, 75, and 150 mg/kg bw/day for 28 days (*n* = 3 animals per group). GAPDH was used as the loading control. Images are representative of three independent experiments. The results are expressed as mean ± SEM and statistically significant differences, as compared with the control (corn oil), are indicated as * *P* < 0.05 and ** *P* < 0.01. Abbreviations: CV, central vein; Bcl-2, B-cell lymphoma 2; Bax, Bcl-2-associated X protein; SD, Sprague Dawley; FTF, flutriafol; GAPDH, glyceraldehyde 3-phosphate dehydrogenase; SEM, standard error of mean.

Rat liver samples were histologically evaluated by hematoxylin and eosin staining (Figure 8A). The liver samples collected from rats treated with FTF at a concentration of 0.25 mg/kg bw/day showed normal liver architecture with no histopathological alteration, compared with the control rats. However, FTF (2.5–150 mg/kg bw/day) treatment induced fatty infiltration in rat liver tissue, and the severity of liver steatosis remarkably increased in the rat livers (75 and 150 mg/kg bw/day FTF) (Figure 8A). In addition, protein expression levels related to crucial mediators of apoptosis (Caspase3 and cleaved Caspase3) were increased in the FTF (75 and 150 mg/kg bw/day)-treated rat livers, compared to control rat livers (Figure 8B). Moreover, the FTF-exposed rat livers had a significantly lower protein expression level of Bcl-2 and a higher protein expression level of Bax (*P* < 0.05).

## 4. Discussion

Pesticides, such as insecticides, herbicides, and fungicides, are widely used to increase agricultural productivity. However, because the excessive use of pesticides results in the accumulation of residues in the soil, water, and food products, humans are routinely exposed to residual pesticides [24]. European Food Safety Authority reported that 11,513 foods (98.6%) of plant and animal origin out of the total of 11,679 foods contained one or more pesticide residues below or equal to the maximum residue level, which is known to consider relatively safe for humans [25]. However, the European Food Safety Authority also announced that the consumer may be exposed to residual FTF of up to 0.5 mg/kg in agricultural commodities [26]. Therefore, we assumed that residual FTF in food products may induce liver toxicity considering the acceptable daily intake and acute reference dose of FTF of 0.01 mg/kg bw/day and 0.05 mg/kg bw/day, respectively [27]. Although the International Union of Pure and Applied Chemistry reported that FTF possesses general toxicity as a liver toxicant [28], there are no detailed data about hepatotoxicity and the underlying toxic mechanisms of FTF in human liver cells. Thus, our study focused on liver toxicity of FTF and the associated cellular mechanisms using human liver cell line HepG2 and an SD rat model.

In our study, cell membrane integrity and cell viability assays were performed for human liver cells. FTF at concentrations of up to 320 µM did not affect cell membrane integrity, whereas 320 and 640 µM FTF significantly decreased cell viability up to 83% and 63% (Figure 1A,B). Therefore, FTF at concentrations up to 320 µM was used for subsequent experiments including cell apoptosis. The liver plays an important role in the detoxification of both endogenous and exogenous compounds. In particular, xenobiotics, such as FTF, absorbed through the digestive system are mainly catalyzed into hydrophilic metabolites by CYP enzymes, the major enzymes of phase I metabolism, in hepatocytes [29]. Previous studies have shown that nuclear receptors, such as aryl hydrocarbon receptor (AHR), constitutive androstane receptor (CAR), and pregnane X receptor (PXR) regulate the transcription of CYP enzymes, and the activated CYP enzymes produced intracellular oxidative stress during the CYP-mediated oxidation reaction cycles [13,30]. However, these generations of ROS during xenobiotic metabolism also affected the activities of nuclear receptor xenosensors, such as AHR, PXR, and CAR, through interaction with the forkhead box, a class O transcription factor [31]. In addition, oxidative stress induced lipid accumulation through sterol regulatory element-binding protein 1c in HepG2 cells [32]. Therefore, we evaluated the correlation between CYP enzymes, ROS generation, and lipid accumulation in FTF-treated human liver cells. Our study showed that FTF induced the accumulation of lipid such as triglycerides and cholesterol in a concentration-dependent manner (Figure 1C,D). In addition, FTF increased the gene expression of CYP enzymes, intracellular ROS production, and MMP loss in human liver cells, whereas pretreatment of cells with NAC reduced the gene expression of CYP enzymes and intracellular ROS (Figure 2 and Figure 3), suggesting that FTF-induced lipid accumulation, which is associated with oxidative stress and gene expression. Similarly, in previous studies, Myclobutanil, a triazole fungicide, treatment to cells increased intracellular lipid accumulation [20]. Additionally, tebuconazole, a triazole compound, induced the expression of CYP1A2 in HepG2 cells, and triazole fungicides (fluconazole, itraconazole, voriconazole, and posaconazole) were biotransformed through the activation of several CYPs, including CYP2C9, CYP2C19, and CYP3A4 [33,34]. Moreover, difenoconazole, a triazole compound, also induced excessive ROS generation and MMP loss in human liver cells [35]. Collectively, our data showed that FTF can induce lipid accumulation with intracellular ROS and gene expression of CYP enzymes, indicating that oxidative stress is one of the major toxic responses in the human liver.

Uncontrolled ROS production is known to activate Nrf2 as the main cellular defense mechanism against oxidative stress [36]. Nrf2 is rapidly degraded by interacting with Kelch-like ECH-associated protein 1 (KEAP1) in the cytosol. However, excessive oxidative stress stabilizes Nrf2 and induces nuclear translocation of Nrf2 [37]. The nuclear translocation of Nrf2 leads to binding with the antioxidant response element (ARE) in the promoter region of the Nrf2 target gene and subsequently activates the transcription of ARE-mediated antioxidant enzymes, such as NQO1, GST, and heme oxygenase-1 [38,39]. In our study, FTF significantly increased the translocation of Nrf2 and mRNA expression levels of NQO1, NQO2, GSTP1, and GSTO2 (Figure 4). Furthermore, the inhibition of oxidative stress using the ROS inhibitor NAC attenuated the observed Nrf2 nuclear translocation and mRNA expression levels of antioxidant enzymes (Figure 4). According to a previous study, miconazole, an antifungal drug, induced ROS generation and Nrf2 activation through the suppression of KEAP1 in human bladder cells [40]. In addition, triazole fungicide (ketoconazole and posaconazole) increased the mRNA expression of Nrf2 and antioxidant enzymes, such as superoxide dismutase2, in HepG2 cells [41]. Therefore, our data demonstrated that FTF induced the activation of Nrf2 and antioxidant enzymes as a defensive mechanism.

The expression of antioxidant enzymes through Nrf2 activation maintains redox homeostasis. Nevertheless, excessive ROS production, which is not controlled by antioxidant enzymes, leads to apoptosis [42]. The apoptosis pathway is classified as both the extrinsic and intrinsic pathways. Extrinsic apoptosis is initiated by the binding of the death ligand to the receptor on the cell membrane, and intrinsic apoptosis is triggered by the release of cytochrome c from the mitochondria through the regulation of mitochondrial permeability [43]. However, apoptosis signaling through the death receptors in hepatocytes cannot sufficiently initiate the extrinsic pathway, and hence, it has been reported that the mitochondria-mediated pathway is mainly activated for the initiation of apoptosis [43]. A previous study revealed that the generation of ROS increased the release of cytochrome c through mitochondrial membrane depolarization, thereby leading to apoptosis [44]. Our study also revealed that FTF impaired mitochondrial membrane function in a concentration-dependent manner (Figure 3C), and a higher concentration of FTF induced the loss of cell viability and increased the apoptosis ratio (Figure 5). However, the decrease in oxidative stress induced by NAC improved the cell viability and apoptosis ratio. In addition, a previous study reported that tebuconazole, which belongs to the family of triazole fungicides, induced oxidative stress in rat kidneys, resulting in apoptosis through the downregulation of Bcl2 and the upregulation of Bax and Caspase3 [45]. Therefore, our data provide evidence that FTF can induce apoptosis in human liver cells through oxidative stress caused by mitochondrial dysfunction.

The experimental dose of FTF (2.5 mg/kg bw/day) in the animal study was chosen based on the maximum consumer risk assessment of FTF (0.5 mg/kg) reported by the European Food Safety Authority, which was translated from humans to rats using body surface area normalization [46]. Furthermore, the lowest level at which an adverse effect was observed (148 mg/kg bw/day), which was reported in a rat toxicity study for 90 days, was considered the maximum dose of FTF [9]. Thus, our selected doses (0.25–150 mg/kg bw/day) included both the potential human exposure dose and previously evaluated maximum dose level for FTF. In our study, rats administered 150 mg/kg bw/day displayed significantly decreased feed intake in the first week (Figure 6A). In fact, triazole compounds have been used as antifungal agents in humans, but it has been reported that the intake of these antifungal agents induced mild to moderate adverse effects, such as gastrointestinal disturbances, abdominal pain, headache, and vomiting [47]. Thus, the loss of feed intake in the first week is considered a mild side effect of FTF intake. In addition, because the variation in organ weight caused by exposure to xenobiotics is an indicator of toxicological alteration [48], the weights of the liver and kidney, as the main metabolic organs for FTF, were measured. The results showed that the administration of 75 and 150 mg/kg bw/day FTF resulted in an increased ratio of liver weight/body weight and kidney weight/body weight, suggesting the hepatotoxicity of FTF (Figure 6B). Subsequently, to investigate further the biological alterations associated with the increase in liver weight and kidney weight, liver and kidney functions were determined using rat blood plasma analysis. The biochemical data demonstrated that the rats treated with 150 mg/kg bw/day FTF showed increased TP and decreased BUN levels (Figure 6C). The upregulation of TP in blood plasma is known to be associated with the onset of fatty liver [49], and the decrease in BUN is an indication of liver dysfunction caused by the impaired urea cycle [50]. Moreover, the impaired urea cycle enzymes are associated with NAFLD [51].

NAFLD is a liver disease related to metabolic disorders [52]. This disease has the economic and social burdens of a total of USD 292 billion in the US [15]. Therefore, we evaluated liver metabolism dysfunction in rats. Humans have different isoform compositions, expression patterns, and metabolic enzymes, compared to rats, and hence, rat CYP1A2 and CYP3A2 were determined as the homologs of human CYP1A2 and CYP3A4 [53]. Consistent with our in vitro data, our data showed that liver samples from rats exposed to FTF had significantly higher mRNA levels of CYP1A2 and CYP3A2 than those from the control rats (Figure 7A). According to a previous report, the activation of CYP enzymes in the liver led to the production of intracellular ROS, and the resulting cellular oxidative stress activateD Nrf2 [13,52]. In contrast, our study showed that the administration of FTF (2.5–150 mg/kg bw/day) decreased the protein expression of Nrf2 in the liver (Figure 7B). These results indicate that FTF leads to an imbalance in redox homeostasis through the loss of Nrf2 activation. In fact, previous studies have reported that oxidative stress accelerated lipid peroxidation with the depletion of antioxidant enzymes, such as GSH and SOD, during the progression of NAFLD, and long-chain fatty acids (palmitate) induced oxidative stress and apoptotic responses in hepatic cells with steatosis [54,55]. Similarly, in this study, histological analysis showed that the administration of FTF to rats caused histopathological alterations in the liver, including steatosis, and the severity was intensified with the increase in the concentration of FTF (Figure 8A). Our data also demonstrated that the administration of FTF resulted in apoptosis through increased expression of Caspase-3 and Bax and decreased Bcl-2 (Figure 8B). In particular, in clinical samples of liver from NAFLD patients, activated Caspase-3 was involved in the apoptosis of hepatocytes and in the progression of NAFLD [56]. In addition, liver injury and the severity of apoptosis were associated with the upregulation of Bax and lower levels of Bcl-2 in NAFLD patients [57]. Therefore, our data demonstrated that FTF can cause liver steatosis, which was mediated by the redox imbalance and apoptosis that occurs during the progression of NAFLD. More importantly, our data demonstrated that the exposure of 2.5 mg/kg bw/day FTF (i.e., equivalent FTF residues level of 0.5 mg/kg in agricultural commodities) to rats resulted in lipid accumulation and redox imbalance in the liver.

In conclusion, our data revealed that FTF can cause lipid accumulation in human liver cells and rat liver through oxidative stress and apoptosis. Although further studies may be needed to identify the role of FTF on NAFLD in humans, our data suggest that chronic exposure of residual FTF to humans may be a potential risk factor for the pathogenesis of NAFLD. According to the current regulation by European Food Safety Authority, the maximum residual limits of FTF are legally tolerated up to 0.4 mg/kg in pome fruit, 0.5 mg/kg in cereals, and 1 mg/kg in grapes [5]. Since our data showed 0.5 mg/kg FTF in agricultural commodities may be a risk of NAFLD development and progression, the regulation about residual FTF needs to be reevaluated.

## Figures and Tables

**Table 1:** Primers for real-time polymerase chain reaction analysis.

Gene ^1^	Primer Sequence 5′‒3′
CYP1A2 (Human)	(F) ATG GCA TTG TCC CAG TCT G (R) TCT GGT GGA CTT TTC AGG C
CYP2C9 (Human)	(F) ATG GAT TCT CTT GTG GTC CTT (R) CAA TCA CTG GGA GAG GAG TG
CYP2C19 (Human)	(F) ATG GAT CCT TTT GTG GTC C (R) TAG GAT ATT TCC AAT CAC TGG G
CYP3A4 (Human)	(F) ATG GCT CTC ATC CCA GAC TTG G (R) CCC TGG AAT TCC AAG CTT CTT
CYP51A1 (Human)	(F) ACC TCT TGT CCA TGC TGC TGA T (R) TGG CAT GCC CAA GGA ATG GA
CYP2E1 (Human)	(F) TGC TGG TGT CCA TGT GGA G (R) CGG GTG AAG GAC TTG GGA AT
NQO1 (Human)	(F) GAC CTC TAT GCC ATG AAC TT (R) TAT AAG CCA GAA CAG ACT CG
NQO2 (Human)	(F) GAG TGG AAA CCC ACG AAG (R) AGC AAA CCG GAA TCG TAG
GSTP1 (Human)	(F) CAG ATC AGG GCC AGA GCT GGA A (R) GGT GAC GCA GGA TGG TAT TGG ACT
GSTO2 (Human)	(F) CTC CTA CTC TCG GGC TTC CAA A (R) AGA AAC AGC TGC GC CTG G
GAPDH (Human)	(F) GAC CCC TTC ATT GAC CTC AAC TAC (R)ATG ACA AGC TTC CCG TTC TCA G
CYP1A2 (Rat)	(F) AAA CCA GTG GCA GGT CAA CCA T (R) TCA CCT TCT CAC TCA GGG TCT TGT
CYP3A2 (Rat)	(F) ACG TTC ACC AGT GGA AGA CTC AAG (R) ACA TCC ATG CTG TAG GCA CCA A
GAPDH (Rat)	(F) TGC CAT CAA CGA CCC CTT CAT T (R) GCA TCA CCC CAT TTG ATG TTA GCG

^1^ CYP, cytochrome P450; NQO, NAD(P)H quinone oxidoreductase; GSTP, glutathione S-transferase P; GSTO, glutathione S-transferase omega, GAPDH, glyceraldehyde 3-phosphate dehydrogenase.
